# The impact and challenges of implementing CTCA according to the 2019 ESC guidelines on chronic coronary syndromes: a survey and projection of CTCA services in the Netherlands

**DOI:** 10.1186/s13244-021-01122-2

**Published:** 2021-12-18

**Authors:** T. P. W. van den Boogert, B. E. P. M. Claessen, S. M. Boekholdt, T. Leiner, R. Vliegenthart, S. F. Schuiling, J. R. Timmer, S. C. A. M. Bekkers, M. Voskuil, H. J. Siebelink, W. van Es, H. J. Lamb, M. Prokop, P. Damman, J. Stoker, H. C. Willems, J. P. Henriques, R. N. Planken

**Affiliations:** 1 Heart centre, Department of Clinical and Experimental Cardiology, Amsterdam Cardiovascular Sciences, Amsterdam UMC, University of Amsterdam, Amsterdam, The Netherlands; 2 Department of Radiology and Nuclear Medicine, Amsterdam Cardiovascular Sciences, Amsterdam UMC, University of Amsterdam, Amsterdam, The Netherlands; 3 Department of Radiology and Nuclear Medicine, Amsterdam UMC, Location AMC, Meibergdreef 9, 1105 AZ Amsterdam, The Netherlands; 4 Department of Cardiology, Noordwest Ziekenhuisgroep, Alkmaar, The Netherlands; 5 Department of Radiology, Utrecht University Medical centre, Utrecht, The Netherlands; 6 Department of Radiology, University Medical centre Groningen, Groningen, The Netherlands; 7 Zorgevaluatie en Gepast Gebruik, Diemen, The Netherlands; 8 Departments of Cardiology, Isala, Zwolle, The Netherlands; 9 Department of Cardiology, Maastricht University Medical centre, Maastricht, The Netherlands; 10 Department of Cardiology, Utrecht University Medical centre, Utrecht, The Netherlands; 11 Department of Cardiology, Leiden University Medical centre, Leiden, The Netherlands; 12 Department of Radiology, St. Antonius Hospital, Nieuwegein, The Netherlands; 13 Department of Radiology, Leiden University Medical centre, Leiden, The Netherlands; 14 Department of Radiology, Nuclear Medicine, and Anatomy, Radboud University Medical centre, Nijmegen, The Netherlands; 15 Department of Cardiology, Radboud University Medical centre, Nijmegen, The Netherlands; 16 Department of Radiology and Nuclear Medicine, Amsterdam Gastroenterology Endocrinology Metabolism, Amsterdam UMC, University of Amsterdam, Amsterdam, The Netherlands; 17 Present Address: Division of Geriatrics, Department of Internal Medicine, Amsterdam UMC, Amsterdam, The Netherlands

**Keywords:** Computed Tomography Angiography, Coronary artery disease, Guidelines

## Abstract

**Background:**

The 2019 ESC-guidelines on chronic coronary syndromes (ESC-CCS) recommend computed tomographic coronary angiography (CTCA) or non-invasive functional imaging instead of exercise ECG as initial test to diagnose obstructive coronary artery disease. Since impact and challenges of these guidelines are unknown, we studied the current utilisation of CTCA-services, status of CTCA-protocols and modeled the expected impact of these guidelines in the Netherlands.

**Methods and results:**

A survey on current practice and CTCA utilisation was disseminated to every Dutch hospital organisation providing outpatient cardiology care and modeled the required CTCA capacity for implementation of the ESC guideline, based on these national figures and expert consensus. Survey response rate was 100% (68/68 hospital organisations). In 2019, 63 hospital organisations provided CTCA-services (93%), CTCA was performed on 99 CTCA-capable CT-scanners, and 37,283 CTCA-examinations were performed. Between the hospital organisations, we found substantial variation considering CTCA indications, CTCA equipment and acquisition and reporting standards. To fully implement the new ESC guideline, our model suggests that 70,000 additional CTCA-examinations would have to be performed in the Netherlands.

**Conclusions:**

Despite high national CTCA-services coverage in the Netherlands, a substantial increase in CTCA capacity is expected to be able to implement the 2019 ESC-CCS recommendations on the use of CTCA. Furthermore, the results of this survey highlight the importance to address variations in image acquisition and to standardise the interpretation and reporting of CTCA, as well as to establish interdisciplinary collaboration and organisational alignment.

## Key points

- Significant increase in CTCA-examinations is required to fully facilitate current European guidelines.
- Guidelines how to perform CTCA should be updated to set higher standards for CTCA equipment, acquisition protocols and image quality.
- CTCA interpretation and reporting should be standardised.

## Introduction

The European Society of Cardiology (ESC) updated guidelines for diagnosis and management of chronic coronary syndromes (ESC-CCS) in coronary artery disease (CAD) [1]. Computed tomography coronary angiography (CTCA) and non-invasive functional imaging for myocardial ischaemia are recommended (Class 1) as initial test for diagnosing CAD instead of exercise electrocardiography [1, 2]. The choice of initial non-invasive imaging test primarily depends on the patient’s pre-test probability of obstructive CAD: CTCA is recommended for those with a lower range pre-test probability, while functional imaging is recommended for those with a higher range pre-test probability. Considering that the majority of patients have a pre-test probability in the lower range, these new guideline recommendations may pose challenges for the availability of CTCA-services [3, 4]. For example, in the Netherlands, the prevalence of CAD is around 800,000 [5]. The prevalence of patients with chest pain and suspected CAD is higher, but the exact number remains unknown. However, we know that 252,449 patients visited a cardiologist for chest pain in 2012. Of the new patients with suspected CAD 61% underwent an exercise ECG [6]. Even if a small proportion of exercise ECGs and invasive coronary angiography (ICA) were to be substituted by CTCA, the demand for CTCA-services is expected to increase substantially. Such a shift would require sufficient numbers of CTCA-capable scanners and competent cardiovascular imaging experts to guarantee national coverage. Moreover, variations in clinical practice need to be addressed to ensure high image quality as well as standardised interpretation and reporting of CTCA findings.

It is currently unknown, what percentage of hospital organisations in Europe provide CTCA-services and how many CTCA-capable scanners are available. There is no overview about indications for which CTCA is deployed, and variations in clinical practice across a country are unknown. The goal of our study is to track these issues in the Netherlands, a country with an advanced healthcare system, as an example. We performed a national survey among members of the Dutch Societies of Radiology (NVvR) and Cardiology (NVVC) in every hospital organisation in the Netherlands to study the current utilisation of CTCA-services and status of CTCA-protocols, and modeled the expected effect of these guidelines on CTCA capacity in the Netherlands. Accordingly, the survey is endorsed by the NVvR and NVVC.

## Methods

### Guideline recommendations considering the use of CTCA

The current ESC-CCS guidelines recommend CTCA for various indications that are listed in Table 1 [1, 7]. In addition, in patients with new onset of heart failure or reduced left ventricular function, ICA or CTCA could be performed to establish the presence and extent of CAD. In accordance with the cited guidelines, this survey did not include patients with suspected acute coronary syndrome.

**Table 1:** Guidelines recommendations for the use of CTCA

Recommendations 2019 ESC Guidelines for the diagnosis and management of chronic coronary syndromes	Class	Level
Risk stratification, preferably using stress imaging or CTCA (if permitted by local expertise and availability), or alternatively exercise stress ECG (if significant exercise can be performed and the ECG is amenable to the identification of ischaemic changes), is recommended in patients with suspected or newly diagnosed CAD	I	B
Non-invasive functional imaging for myocardial ischaemia or CTCA is recommended as the initial test to diagnose CAD in symptomatic patients in whom obstructive CAD cannot be excluded by clinical assessment alone	I	B
ICA or CTCA is recommended in patients with characteristic episodic resting angina and ST-segment changes, which resolve with nitrates and/or calcium antagonists, to determine the extent of underlying coronary disease	I	C
Coronary CTA should be considered as an alternative to coronary angiography before valve intervention in patients with severe valvular heart disease and low probability of CAD	IIa	C

The mentioned ESC guideline recommendations are class I (recommended or indicated) and class IIa (should be considered). The level of evidence is: A, derived from multiple randomised clinical trials or meta-analyses, B, derived from a single randomised clinical trial or large non-randomised studies, C, is a consensus of opinion of the experts and/or small studies, retrospective studies, registries

### The Dutch health-care setting

Outpatient cardiology care was provided by 68 hospital organisations (107 hospital locations) in the Netherlands in 2019 [8]. There are 16 hospital organisations that perform cardiac surgery and percutaneous coronary interventions (PCI) (eight university hospitals and eight large non-university hospitals). Beside these 16 cardiothoracic surgical centres, 14 other hospitals perform PCI (off-site PCI centres). The other hospitals do not perform PCI (non-PCI centres). Of these 38 non-PCI centres, 36 do perform diagnostic ICA.

### Survey design

An electronic survey was designed (LimeSurvey, Hamburg, Germany) and reviewed afterwards by the cardiac-imaging sections of the NVvR and NVVC and send to cardiac imaging specialist of all 68 hospital organisations in December 2019. Non-responders were reminded and eventually contacted personally to fill in the survey.

With the survey, we aimed to answer four principal questions: 1) what is the current provision and utilisation of CTCA services within the Dutch health care system? 2) For which indications is CTCA used and practiced and what are the differences between hospitals? 3) What is the current status of CTCA-protocols regarding patient preparation, image acquisition protocols and standard operating procedures (SOPs) for CTCA acquisition, interpretation and CTCA reporting? 4) What is the modeled necessity for CTCA in the Netherlands when implementing 2019 ESC-CCS guidelines?

### Data analysis

Statistical analyses were performed by using R software version 3.5.1 (R Foundation for Statistical Computing, Vienna, Austria) [9]. Counts were presented as numbers and continuous variables as means with corresponding standard deviations or medians with interquartile ranges. Categorical variables were presented as frequencies and percentages. Hospitals were divided by type (cardiothoracic surgical centres, PCI centres and non-PCI centres).

For modeling the necessity of CTCA-services, we based the expected change in diagnostic tests on the recommendations in the 2019 ESC-CCS guidelines [1]. Numbers and figures were based on historical trend data of patients with suspected CAD in the Netherlands, as published by the Dutch National Health Care Institute in 2017 [6]. We projected the historical trend data to approach current numbers, using linear curve fitting. Based on Dutch literature, the conversion factor to approach the number of patients for 2020 was 1.15 [6]. We modeled potential scenarios with a 50%, 75% and 100% implementation rate of the recommended diagnostic algorithm.

## Results

### Survey responses and CTCA providing hospitals

**Fig. 1:**
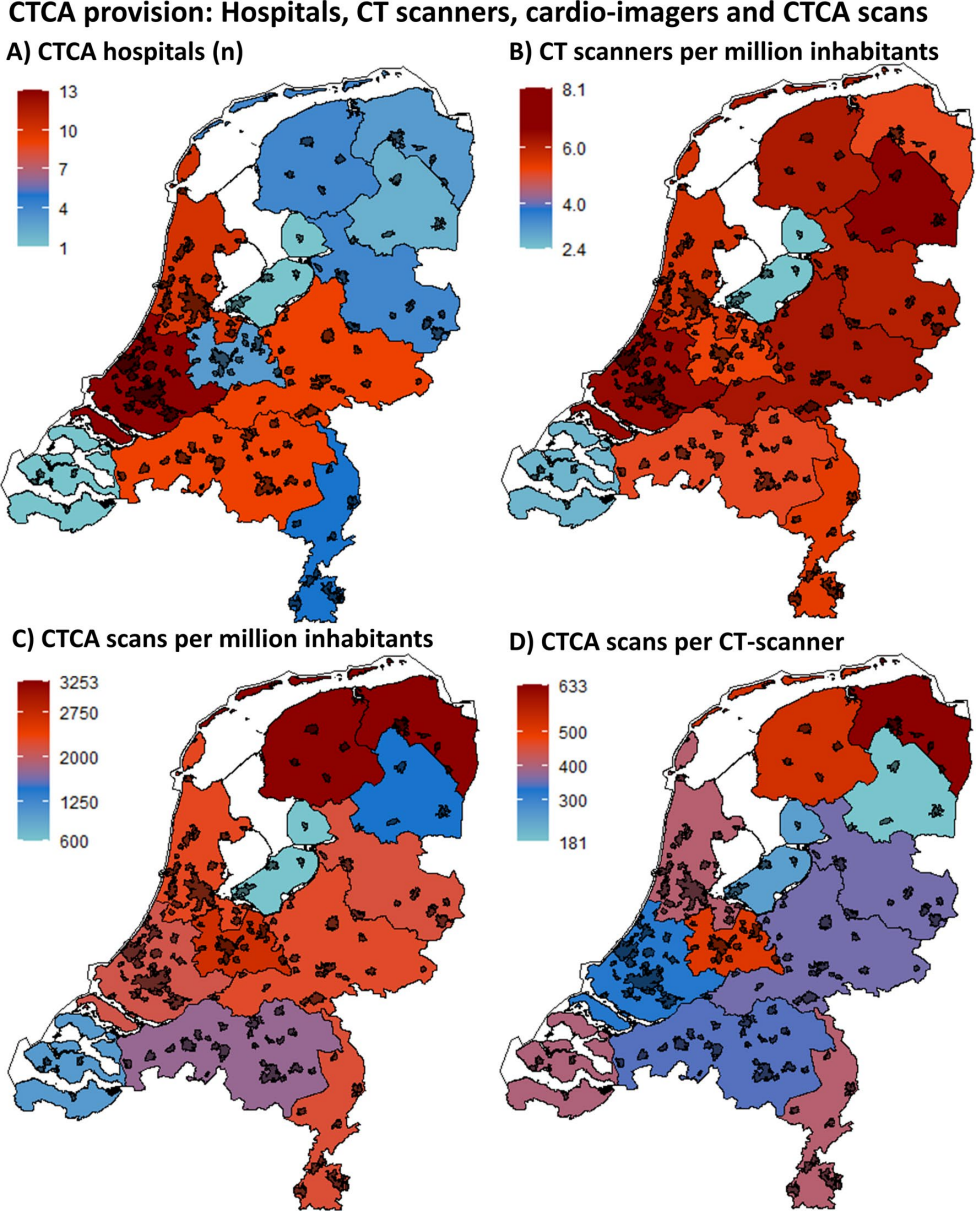
(**a**) The number of hospitals that perform CTCA, (**b**) the number of CTCA-capable CT-scanners per million inhabitants, (**c**) the number of CTCA-examinations per million inhabitants, (**d**) the number of CTCA-examinations per CT-scanner. The black coloured areas represent densely populated urban areas

All 68 (100%) hospital organisations in the Netherlands responded to the survey, of which five (7.4%) responded not to perform CTCA. Of these five non-CTCA hospitals are two are PCI-centres and three non-PCI centres, all five perform ICA. Most hospitals providing CTCA-services are located in highly populated provinces (Fig. 1a, Table 2).

**Table 2:** CTCA provision and utilisation

Province	Inhabitants (*x* million)	Hospitals all (CTCA)	CT-scanners *n* (per million)	CTCA examinations	CTCA per scanner
Groningen	0.58	3 (3)	3 (5.1)	1900	633
Friesland	0.65	4 (4)	4 (6.2)	2100	525
Drenthe	0.49	2 (2)	4 (8.1)	725	104
Overijssel	1.16	4 (3)	7 (6.1)	2508	177
Flevoland	0.42	1 (1)	1 (2.4)	250	250
Gelderland	2.07	9 (9)	13 (6.3)	4650	341
Utrecht	1.34	4 (3)	7 (5.2)	3550	507
Noord-Holland	2.85	12 (10)	16 (5.6)	6525	343
Zuid-Holland	3.67	14 (13)	24 (6.5)	7751	271
Zeeland	0.38	2 (1)	1 (2.6)	400	400
Noord-Brabant	2.54	9 (9)	13 (5.1)	4474	352
Limburg	1.12	5 (5)	6 (5.4)	2450	408
Netherlands	17.3	69 (63)	99	37,283	364

CTCA provision and utilisation, listed per province and for the entire country. *n* number, *CT* computed tomography, *CTCA* computed tomography coronary angiography

### What is the current provision and utilisation of CTCA-services within the Dutch health care system?

A total of 99 CTCA-capable CT-scanners are available in 63 hospitals ranging from 1 to 4 scanners per hospital, with 36 using 1 CT-scanner, 19 hospitals using 2 CT-scanners, 7 hospitals using 3 CT-scanners and 1 hospital using 4 scanners. Per million inhabitants, the mean number of CTCA-capable CT-scanners is 5.4 with a standard deviation of 1.6 and a range of 2.4–8.1 per province (Fig. 1b).

**Fig. 2:**
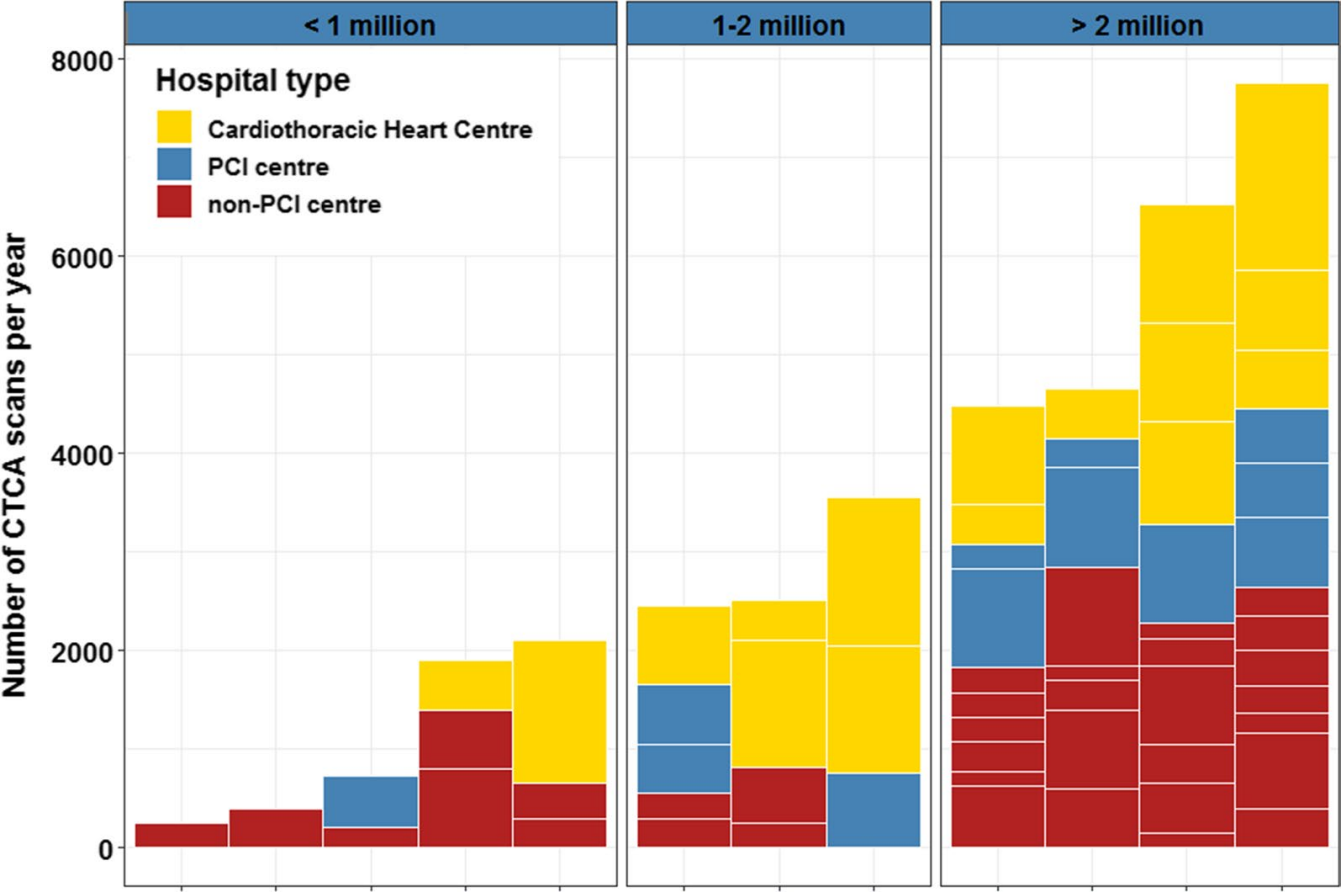
The number of CTCA-examinations per hospital type. The number of CTCA scans is plotted on the y-axis per hospital, as coloured boxes. The stacked boxes correspond with the cumulative number of CTCA-examinations per province (*x*-axis). The provinces are grouped, according to the number of inhabitants: < 1 million inhabitants (left frame), 1–2 million inhabitants (middle frame) and > 2 million inhabitants (right frame). The colour of the boxes correspond with the type of hospital, as listed in the legend

Across the Netherlands, 37,283 CTCA-examinations were performed in 2019, with an average of 2,085 per million inhabitants, ranging from 600 to 3,253 per province (Fig. 1c). An average of 391 (181–633) CTCA-examinations were performed per CT-scanner (Fig. 1d) and an average of 512 (150–1900) per hospital (Fig. 2). The cardiothoracic surgical centres had the highest mean number of CTCA-examinations per year (981 per centre, 15,700 total), followed by PCI centres (552 per centre, 7,730 total) and the non-PCI centres (355 per centre, 13,853 total).

Most CT-scanners are not dedicated CTCA-scanners and also used for non-CTCA indications (i.e., chest, abdominal, musculoskeletal, neuro, paediatric and intervention). Therefore, this number indicates only a proportion of the total scan time per scanner.

### For which indications is CTCA used and practiced in the hospitals in the Netherlands and what are the differences between types of hospitals?

**Fig. 3:**
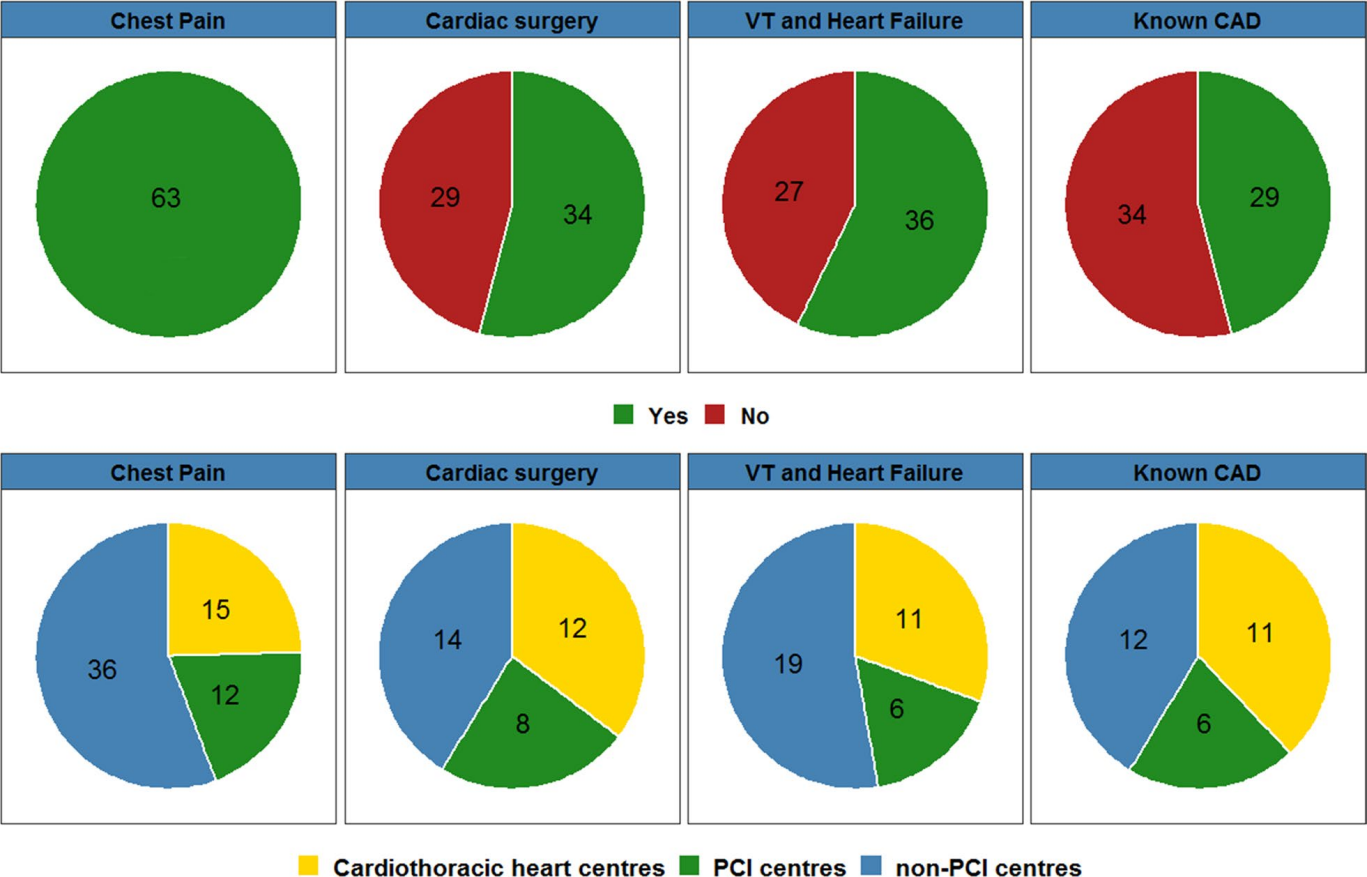
**First row** Pie charts showing the number of hospitals that perform CTCA for the indications: Chest pain, non-coronary cardiac surgery, ventricular tachycardia (VT) or heart failure and known CAD. **Second row** Differentiation in hospitals type of the hospitals that perform CTCA for the corresponding indication. The number of hospitals is shown in the pie slices

All 63 CTCA performing hospital organisations apply CTCA in patients with suspected CAD (Fig. 3) of which 13 (20.6%) only perform CTCA in patients with low probability of CAD, 44 (69.9%) in patients with low or intermediate probability, and 6 (9.5%) in patients with low, intermediate or high probability.

Besides chest pain indications, CTCA is used to exclude CAD in patients awaiting non-coronary cardiac surgery (34 hospitals 54.0%), in patients with ventricular tachycardia or heart failure (38 hospitals, 60.3%), and in patients with known CAD (30 hospitals, 47.6%) (Fig. 3).

### What is the current status of CTCA-protocols (Patient preparation, image acquisition protocols and SOP’s for CTCA interpretation and CTCA reporting) in the hospitals in the Netherlands?

#### CT-scanners and Image acquisition

For image acquisition, all hospitals perform CTCA on CT-scanners with 64 or more slices, meeting the minimum detector requirement set by the Society of Cardiovascular Computed Tomography (SCCT) [10]. A total of 45 hospitals (71.4%) perform CTCA on a CT-scanner with 256 slices or more, of which 15 hospitals (23.8%) perform CTCA on high-end scanners with 320 or more slices. The other 18 hospitals perform CTCA on CT-scanners with 128 slices (*n* = 7, 11.1%) or 64–128 slices (*n* = 11, 17.5%). Sublingual nitro-glycerine is administered in 56 (88.9%) of hospitals. For image acquisition, 22 hospital organisations used a high pitch spiral protocol, 39 hospital organisations used a prospective ECG gated protocol, and 2 hospitals used a retrospective gated protocol. Beta-blocker medication is administered in all hospitals, either in tablet form before or intravenously during CTCA-examination.

#### CTCA reporting, interpretation and communication

For CTCA reporting, interpretation and communication, there are large differences between the Dutch hospitals. Firstly, the majority of, but not all, hospitals use a standard report for CTCA reporting. Of the 48 hospitals (76.2% of all hospitals that provide CTCA) that do use a standard report, 9 hospitals (18.8%) use the Coronary Artery Disease Reporting And Data System (CAD-RADS), of which 6 (66.7%) also use CAD-RADS in their clinical protocol to guide further patient management [11]. Secondly, there is substantial variation in interpretation of CTCA-findings. Cardiac imagers in 27 hospitals (42.9%) use a cut-off value of ≥ 50% diameter stenosis to indicate a clinically relevant lesion, whereas in 31 (49.2%) us a cut-off value of ≥ 70%. In the remaining 5 hospitals (7.9%), both cut-off values are used. These cut-off values are determined by visual estimation of the lesion (eyeballing) in 19 hospitals (30.2%) and by electronic measurements in 44 hospitals (69.8%). Thirdly, there is no standard procedure for the communication and discussion of CTCA-findings. In 7 hospitals (11.1%), findings are discussed in a heart team (consisting of a cardiologist, cardio-thoracic surgeon and in three hospitals a radiologist), and 21 (33.3%) hospitals use another form of multidisciplinary consultation. In 24 hospitals, there is some form of case discussion and in 11 hospitals (17.5%), there is no further communication of CTCA-findings apart from the CTCA-report.

#### Downstream diagnostic testing

**Fig. 4:**
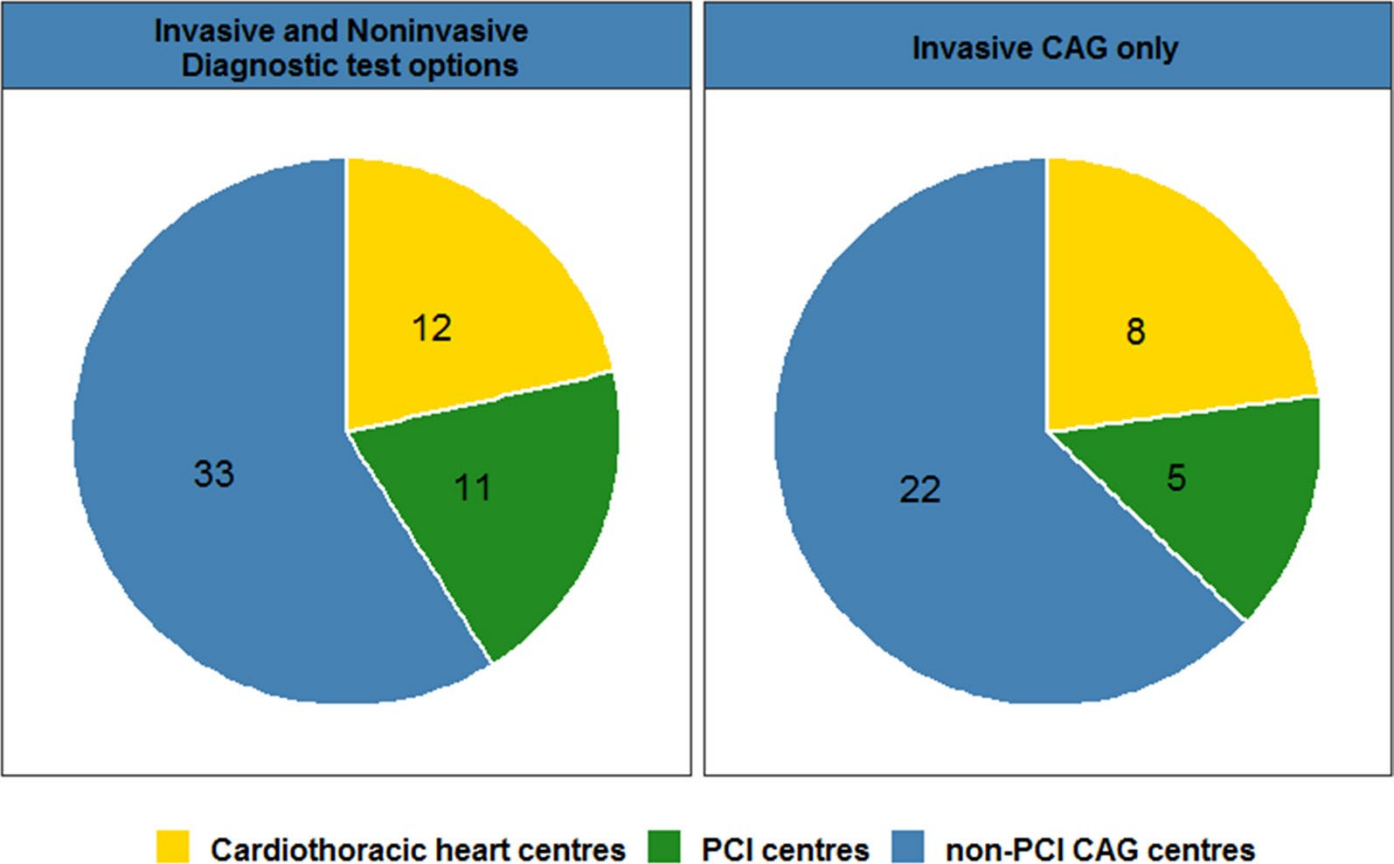
Downstream diagnostic tests after CTCA with significant stenosis, differentiated between hospital types. The number of hospitals is shown in the pie slices. * There was one hospital that only performed non-invasive diagnostics, which was a non-PCI centre

If a clinically relevant stenosis is reported, hospitals use a variety of diagnostic tests for further evaluation. Of the 63 hospital organisations that perform CTCA, 30 (47.6%) use ICA or non-invasive functional testing such as perfusion MRI or nuclear testing (Fig. 4). In 33 hospitals (52.4%), only ICA is used after CTCA. The majority of these latter hospitals are non-PCI centres (Fig. 4). In these hospitals, an additional revascularisation procedure cannot be performed if indicated. However, from the results of our survey, it is not clear if these non-PCI centres will refer patients to a PCI-centre for ICA, or that these patients will undergo diagnostic ICA in non-PCI centre followed by ICA and revascularisation in a PCI centre.

#### Future perspective: modeled necessity for CTCA-services

**Fig. 5:**
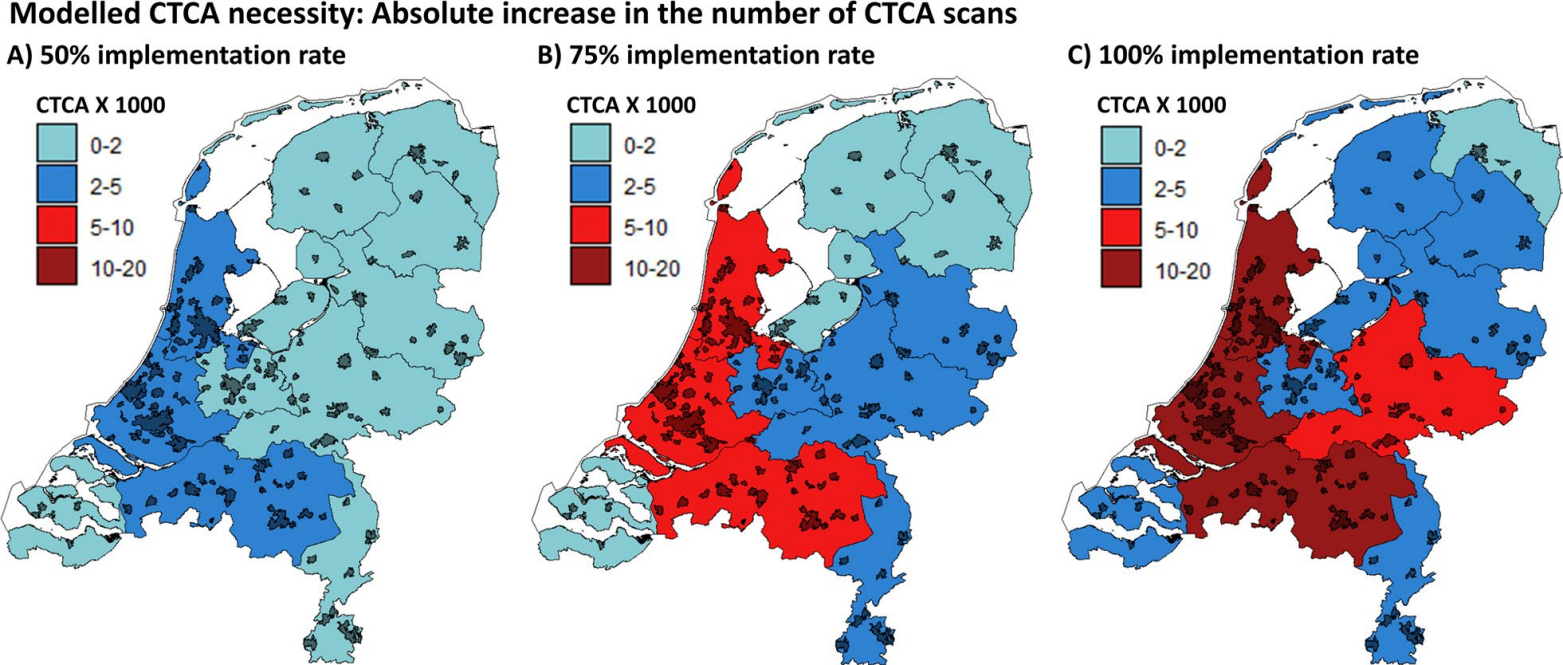
Absolute number increase in annual CTCA scans per province for an implementation rate of the current ESC guidelines of 50% (**a**), 75% (**b**) and 100% (**c**)

A total of 252,449 patients visited a cardiologist for chest pain in the Netherlands in 2012, which is modeled to be 290,000 in 2020 (using the conversion factor of 1.15) [6]. This number includes ~ 50% patients with acute coronary syndrome or patients with known (obstructive) CAD and 50% new patients with suspected CAD. Based on the 2019 ESC-CCS recommendations, only the patients with low to intermediate pre-test probability would require initial CTCA (75% of total). Therefore, the total population that would require CTCA is projected to be 106,450 patients per year in the Netherlands [1]. Currently, a total of 37,283 CTCA-examinations are performed in the Netherlands. Accordingly, to reach a 50% implementation rate of the current clinical guidelines, the total number of CTCA-examinations that need to be performed annually is 53,225 (50.0% of 106,450). Therefore, the expected increase in annual CTCA-examinations will be approximately 16,000 (53,225 minus 37,283). Across the provinces, this number ranges from 0 to 3,550 (Fig. 5a). To reach a 75% implementation rate, the expected increase in annual CTCA-examinations will be ~ 42,500 (79,838 minus 37,283), with a range from 872 to 9,201 across the provinces (Fig. 5b). The maximum increase in annual CTCA-examinations is projected to be ~ 70,000 (106,450 minus 37,283) at a 100% implementation rate, with a range of 1796–14,852 examinations across the provinces (Fig. 5c).

## Discussion

This national CTCA-survey in the Netherlands provides an overview of the current and modeled CTCA provision and utilisation. Sixty-three Dutch hospital organisations (92.6% of all hospital organisations) provide CTCA-services on a total of 99 CTCA-capable CT-scanners with a total of 37,283 CTCA-examinations per year. There is substantial variation between hospitals considering CTCA indications, available CTCA equipment and applied CTCA-protocols. Implementation of the 2019 ESC-CCS guideline will substantially increase the demand for high quality CTCA-examinations with a high impact on healthcare systems.

However, it should be noted that according to 2019 ESC-CSS guidelines, no type of cardiac imaging should take place before appropriate cardiological assessment, as chest complaints may develop in various clinical contexts. However, if diagnostic work-up is indicated, CTCA or non-invasive functional imaging for myocardial ischaemia is recommended as the initial tests [1]. Clinical implementation of these recommendations will affect a substantial proportion of cardiac healthcare and is likely to lead to a shift away from exercise ECG and the other traditional tests for CAD assessment. This may pose challenges in organisation of CAD-related healthcare and impose an extra burden on CT capacity and cardiac imagers. However, the initial deployment of CTCA or non-invasive functional imaging for myocardial ischaemia may improve the prognosis of patients. The use of CTCA as initial test in patients with suspected CAD has been shown to better guide preventive medical therapy, which subsequently led to a reduction in cardiovascular death or non-fatal myocardial infarction in the SCOT-HEART trial [1, 12, 13]. Additionally, the improved diagnostic accuracy may result in early diagnosis and selective treatment of CAD that may prove more cost-effective compared to traditional diagnostic algorithms, thereby considering the direct costs of the procedure and potential downstream cost reduction through preventive medical therapy. However, additional randomised and sufficiently powered studies are needed to make solid statements about cost-effectiveness and improved prognosis.

Challenges for CTCA implementation that need to be taken into account comprise general factors such as organisational culture, networks, communication, leadership, evaluation and feedback, as well as scarce resources such as time, financial resources and education and training of staff [14, 15]. Moreover, there are specific barriers for the implementation of CTCA, consisting of availability of CTCA-services to provide national coverage with sufficient CTCA-capable CT-scanners and competent cardiovascular imaging experts to facilitate the number of required CTCA-examinations. Almost all hospital organisations in the Netherlands provide CTCA-services and have performed 37,283 examinations last year. However, the projected increase in CTCA-services will call for additional investments in CTCA-capable CT-scanners, cardiovascular imaging experts and CT technicians. Secondly, there are challenges on a level of national health care system organisation and health care reimbursements. Accordingly, as implementation of initial CTCA for patients with CCS will divert patient flows away from traditional testing, intensified collaboration between the departments of cardiology and radiology is recommended.

Another challenge, specific for CTCA is the difference in image quality and the absence of standard operation procedures for CTCA-examinations across hospitals. To address this problem, the SCCT has reported guidelines for the performance and acquisition of coronary computed tomographic angiography [10]. Although all hospital organisations perform CTCA on CT-scanners that meet minimum standards (64 slice detector width), variation in acquisition protocols among hospitals remains substantial, and this is inextricably linked to differences in image quality and clinical utility. For example, recommended beta-blocker medication is administered in all hospitals whereas recommended nitro-glycerine is not administered in a substantial percentage of hospitals (11.1%) [10]. Adequate CTCA equipment and protocols will increase the accuracy of stenosis evaluation, thereby reducing the number of a false positive CTCA-examinations [16–19]. These factors also will affect the accuracy of new frontiers in CTCA such as CT-FFR and CT myocardial perfusion [20, 21]. Besides image quality, CTCA equipment and protocols have major effects on radiation dose of CTCA which should also be taken into account [22]. In the Netherlands, national coverage of modern CT-scanners is already high, with 71.4% CT-scanner that have 256 slices or more.

Lastly, there is increasing demand for standardised reporting of CTCA findings. The CAD-RADS or ESCR smart reporting tool are standardised communication and reporting methods that link CTCA findings to further management recommendations and provide additional prognostic information of future CAD events [11, 23, 24]. Although the majority of hospital organisations (76.2%) use a standard report, only a minority (14.3%) used the CAD-RADS reporting system. Furthermore, different hospitals use different cut-off values for a clinically relevant coronary stenosis by either stenosis measurement or visual assessment. This variation in clinical practice is already recognised by the NVvR and NVVC and resulted in a national initiative that aims to optimise the quality of CTCA and reduce the variation in clinical practice by developing uniform image acquisition, post-processing, interpretation and reporting protocols.

The British Society of Cardiovascular Imaging assessed the provision and capability of CTCA within the UK in 2016 and described similar findings with modeled increase in CTCA production of ~ 700% [25]. We, however, report a lower modeled increase in CTCA production of ~ 300%. These differences might be explained by the different recommendations by the NICE for the UK and the ESC for the Netherlands. The 2016 British NICE guidelines recommend CTCA for all chest pain patients, whereas the current ESC 2019 recommend either CTCA or non-invasive functional imaging for myocardial ischaemia as initial tests and only recommend diagnostic testing in patients with a pre-test probability of > 15% [1, 26]. Besides, the UK performed a mean number of 592.5 CTCA-examinations per million inhabitants in 2016 which is substantially lower compared with 2085 CTCA-examinations per million inhabitants in the Netherlands in 2019. Compared to the British model, we also included patients in our prediction model with an indication for coronary evaluation in the diagnostic work-up for ventricular tachycardia, cardiomyopathy and heart failure and in the work-up for non-coronary cardiac surgical and transcatheter procedures. This group represents a significant number of patients in which CTCA is able to safely exclude obstructive CAD [16, 17].

## Limitations

The current survey explored the provision and utilisation of CTCA-services in the Netherlands. With this survey, we cannot evaluate how hospitals use diagnostic modalities for individual patients and to what extent the recent guidelines are already followed. A patient specific assessment is necessary to decide which diagnostic work-up is indicated for each individual patient, depending on specific characteristics such as heart rhythm and frequency, kidney function, implanted cardiac devices, obesity. Secondly, the results of this survey only comprised the total annual number of performed CTCA-examinations. Consequently, we are not able to differentiate between the numbers of performed CTCA-examinations per indication and are unable to report numbers for different pre test probability categories, numbers of (preceding) coronary artery calcium score scans or presence of risk-modifiers. Thirdly, we did not include information on the number of non-invasive and invasive downstream diagnostic tests after CTCA (i.e., perfusion MRI, Single-photon emission computed tomography, ICA). Adding this information would have been valuable to better capture the current use of CTCA in clinical practice and interplay with other tests (coronary artery calcium score, non-invasive functional test and invasive test). Furthermore, this information would be valuable to better understand differences in diagnostic approaches between hospitals.

Lastly, we did not inquire CTCA radiation exposure. Despite a substantial reduction in radiation exposure (78% from 2007 to 2017), a large inter-site variation in radiation exposure persists (factor 37, 57–2090 mGy*cm) [22]. Information about CTCA radiation exposures in The Netherlands in 2018 would add valuable information about the potential of protocol optimization for further radiation exposure reduction in the future.

## Conclusions

Despite high national CTCA-services coverage in the Netherlands, a substantial increase in CTCA capacity is expected to be able to implement the 2019 ESC-CCS recommendations on the use of CTCA. Furthermore, the results of this survey highlight the importance to address variations in image quality and to standardise the interpretation and reporting of CTCA, as well as to establish interdisciplinary collaboration and organisational alignment.

## Data Availability

The datasets used and/or analysed during the current study are available from the corresponding author on reasonable request.

## Abbreviations

CABG: Coronary artery bypass grafting
CAD: Coronary artery disease
CAD-RADS: Coronary artery disease reporting and data system
CTCA: Computed tomography coronary angiography
ESC: European society of cardiology
Exercise ECG: Exercise electrocardiogram
ICA: Invasive coronary angiography
MRI: Magnetic resonance imaging
NVVC: Dutch society of cardiology
NVvR: Dutch society of radiology
PCI: Percutaneous coronary intervention
SCCT: Society of cardiovascular computed tomography
SOP: Standard operating procedure

## References

[1] Knuuti J, Wijns W, Achenbach S (2020). 2019 ESC guidelines for the diagnosis and management of chronic coronary syndromes. Eur Heart J.

[2] Montalescot G, Sechtem U, Achenbach S (2013). 2013 ESC guidelines on the management of stable coronary artery disease: the task force on the management of stable coronary artery disease of the European Society of Cardiology. Eur Heart J.

[3] Bing R, Singh T, Dweck MR (2020). Validation of European Society of Cardiology pre-test probabilities for obstructive coronary artery disease in suspected stable angina. Eur Heart J Qual Care Clin Outcomes.

[4] Juarez-Orozco LE, Saraste A, Capodanno D (2019). Impact of a decreasing pre-test probability on the performance of diagnostic tests for coronary artery disease. Eur Heart J Cardiovasc Imaging.

[5] Leening MJG, Siregar S, Vaartjes I (2014). Heart disease in the Netherlands: A quantitative update. Neth Hear J.

[6] Nederland Z (2017) Zinnige Zorg Room for improvement analysis: Chest pain (Suspected) Stable Angina Pectoris. https://english.zorginstituutnederland.nl/publications/reports/2017/12/17/room-for-improvement-analysis-chest-pain-suspected-stable-angina-pectoris---zinnige-zorg

[7] Baumgartner H, Falk V, Bax JJ (2017). 2017 ESC/EACTS guidelines for the management of valvular heart disease. Eur Heart J.

[8] (NIVEL) MMJN, (RIVM) MJJCP, A.M. Gommer red. (RIVM), (RIVM) FFJ red (2018) Volksgezondheid en zorg. In: 01–05–2020

[9] R Development Core Team R (2011) R: A language and environment for statistical computing. R Foundation for Statistical Computing, Vienna. http://www.R-project.org

[10] Abbara S, Blanke P, Maroules CD (2016). SCCT guidelines for the performance and acquisition of coronary computed tomographic angiography: a report of the society of cardiovascular computed tomography guidelines committee: endorsed by the North American Society for Cardiovascular Imaging (NASCI). J Cardiovasc Comput Tomogr.

[11] Cury RC, Abbara S, Achenbach S (2016). CAD-RADSTMCoronary artery disease—reporting and data system. an expert consensus document of the society of cardiovascular computed tomography (SCCT), the American College of Radiology (ACR) and the North American Society for Cardiovascular Imaging (NASCI). J Cardiovasc Comput Tomogr.

[12] Newby D, Williams M, Hunter A (2015). CT coronary angiography in patients with suspected angina due to coronary heart disease (SCOT-HEART): an open-label, parallel-group, multicentre trial. Lancet.

[13] Investigators TS-H (2018) Coronary CT angiography and 5-year risk of myocardial infarction. N Engl J Med 379:924–933. 10.1056/NEJMoa1805971 10.1056/NEJMoa1805971 30145934

[14] Bach-mortensen AM, Lange BCL, Montgomery P (2018). Barriers and facilitators to implementing evidence-based interventions among third sector organisations : a systematic review. Implement Sci.

[15] Li S, Jeffs L, Barwick M, Stevens B (2018). Organizational contextual features that influence the implementation of evidence- based practices across healthcare settings: a systematic integrative review. Syst Rev.

[16] van den Boogert TPW, Vendrik J, Claessen BEPM (2018). CTCA for detection of significant coronary artery disease in routine TAVI work-up: a systematic review and meta-analysis. Neth Heart J.

[17] Opolski MP, Staruch AD, Jakubczyk M (2016). CT Angiography for the detection of coronary artery Stenoses in patients referred for cardiac valve surgery: systematic review and meta-analysis. JACC Cardiovasc Imaging.

[18] Li P, Xu L, Yang L (2018). Blooming artifact reduction in coronary artery calcification by a new de-blooming algorithm: initial study. Sci Rep.

[19] Fei X, Du X, Yang Q (2008). 64-MDCT coronary angiography: phantom study of effects of vascular attenuation on detection of coronary stenosis. AJR Am J Roentgenol.

[20] Ko BS, Cameron JD, Munnur RK (2017). Noninvasive CT-derived FFR based on structural and fluid analysis: a comparison with invasive FFR for detection of functionally significant stenosis. JACC Cardiovasc Imaging.

[21] Seitun S, De Lorenzi C, Cademartiri F (2018). CT myocardial perfusion imaging: a new frontier in cardiac imaging. Biomed Res Int.

[22] Stocker TJ, Deseive S, Leipsic J (2018). Reduction in radiation exposure in cardiovascular computed tomography imaging: Results from the PROspective multicenter registry on radiaTion dose Estimates of cardiac CT angIOgraphy in daily practice in 2017 (PROTECTION VI). Eur Heart J.

[23] Bittner DO, Mayrhofer T, Budoff M (2020). Prognostic value of coronary CTA in stable chest pain: CAD-RADS, CAC, and cardiovascular events in PROMISE. Cardiovasc Imaging.

[24] Williams MC, Moss A, Dweck M (2020). Standardized reporting systems for computed tomography coronary angiography and calcium scoring: a real-world validation of CAD-RADS and CAC-DRS in patients with stable chest pain. J Cardiovasc Comput Tomogr.

[25] Dreisbach JG, Nicol ED, Roobottom CA, Padley S, Roditi G (2018). Challenges in delivering computed tomography coronary angiography as the first-line test for stable chest pain. Heart.

[26] Moss AJ, Williams MC, Newby DE, Nicol ED (2017). The updated NICE guidelines: cardiac CT as the first-line test for coronary artery disease. Curr Cardiovasc Imaging Rep.

